# Spontaneous Unilateral Chiari I Secondary to Acquired Tonsillar Hypertrophy/Pseudomass With Syringomyelia in a Juvenile With Progressive Scoliosis

**DOI:** 10.5435/JAAOSGlobal-D-22-00111

**Published:** 2023-08-21

**Authors:** Cade A. Morris, D. Alex Forrester, Rosemarie Zanabrie, William Puffinbarger, Neil Borden

**Affiliations:** From the Department of Orthopedic Surgery and Rehabilitation, Oklahoma University Medical Center, Oklahoma City, OK (Dr. Forrester and Dr. Puffinbarger); and the Department of Radiological Sciences, University of Oklahoma College of Medicine, Oklahoma City, OK (Dr. Zanabrie and Dr. Borden).

## Abstract

Chiari type 1 malformation (CM-1) is a structural defect of the central nervous system in which part of the cerebellar tonsils descend below the level of the foramen magnum, sometimes with associated syringomyelia. Although Chiari malformations were traditionally believed to be congenital, several cases of acquired CM-1 with syringomyelia have been reported. Usually associated with repeat lumbar puncture, increased intracranial pressure, and craniocephalic disproportion, CM-1 in the absence of an underlying etiology is rare. We report a rare case of spontaneous idiopathic tonsillar hypertrophy causing unilateral CM-1 with syringomyelia associated with progressive scoliosis in a juvenile with a previously normal neonatal MRI brain and no known underlying pathology. A 9-year-old boy was found to have scoliosis at a routine well-child visit with progression indicated on radiographs 4 months later. Whole spine MRI was performed and showed a new CM-1 with globular, mass-like configuration of the descended right tonsil with otherwise normal tonsillar characteristics. Surgical decompression via suboccipital craniectomy and C1 laminectomy with duraplasty was performed with improvement illustrated on repeat MRI 3 months postoperatively. This rare case emphasizes the importance of routine MRI spine early in select patients with idiopathic scoliosis and illustrates the favorable outcomes noted after decompressive craniectomy.

Chiari malformation has been described as a structural defect in which part of the cerebellar tonsils descend below the level of the foramen magnum.^1^ In 30 to 70% of cases, Chiari type 1 malformation (CM-1) has associated syringomyelia.^2-4^ Chiari malformations were traditionally believed to be congenital anomalies. However, several cases of acquired Chiari malformation with associated syringomyelia have been reported. These include Chiari malformation secondary to spontaneous cerebrospinal fluid (CSF) leakage, repeated lumbar puncture, mass-occupying lesions (with associated increased intracranial pressure), and cranioencephalic disproportion.^1,4-6^ Isolated cerebellar tonsil enlargement has been seen in cases of infiltrative neoplasm, infection, and inflammation.^7,8^ We report a rare case of spontaneous idiopathic tonsillar hypertrophy causing unilateral Chiari malformation with syringomyelia associated with progressive scoliosis in a juvenile with a previously normal neonatal MRI brain and no known underlying pathology. An extensive literature search was performed, and to the authors' knowledge, such a case has not been reported.

## Case Report

**Figure 1:**
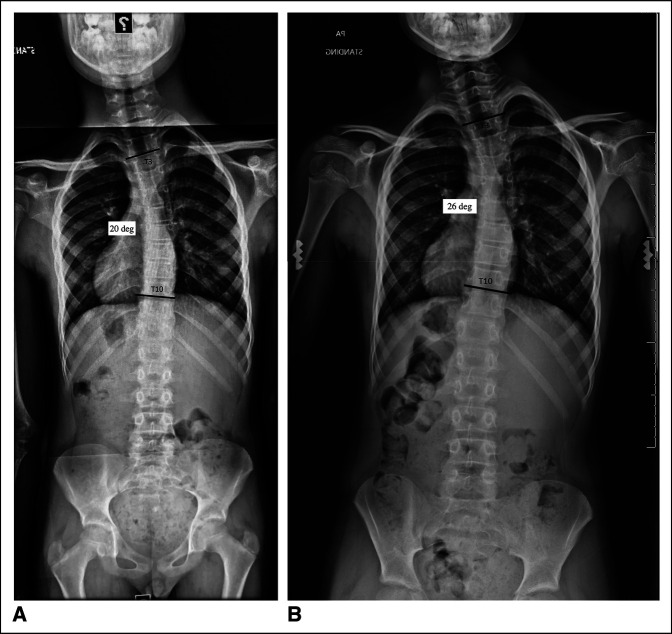
**A,** Upright standing radiograph from scoliosis series shows a midthoracic dextroscoliosis (Cobb Angle 20°). **B,** Upright standing radiograph from scoliosis series shows progression of a midthoracic dextroscoliosis (Cobb Angle 26°).

**Figure 2:**
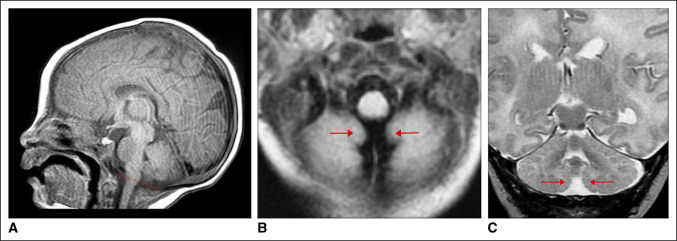
**A,** Sagittal T1 weighted MRI shows symmetrically normal position of the cerebellar tonsils above the foramen magnum (McCrae Line) with no compression of the cervicomedullary junction. **B,** Axial T1 weighted MRI shows symmetrically normal cerebellar tonsils (red arrows) lying above the foramen magnum with symmetrically normal signal intensity. **C,** Coronal T2 weighted MRI shows symmetrically normal cerebellar tonsils (red arrows) lying above the foramen magnum with symmetrically normal signal intensity.

A 9-year-old boy was referred to our institution after a routine well-child visit was notable for scoliosis (Figure 1A). On questioning, the child's adoptive parents admitted to noticing some evidence of a curve over a 1-year period before presentation. The patient had no pain, weakness, numbness, headaches, or other complaints at the time of the visit. His medical history was notable for delayed-start and neonatal hypotonia requiring admission to the neonatal intensive care unit after birth. He was evaluated after birth and followed up through his early life by a geneticist and a neurologist. Extensive genetic testing was found to be normal. Within a week of birth, he was evaluated with brain MRI, which was normal. The cerebellar tonsils were symmetrically normal in size, configuration, and signal intensity (Figure 2, A–C).

On physical examination, he was healthy appearing with slight flank and shoulder asymmetry. He had rotational asymmetry on Adam forward bending test. His strength was 5/5 from C5-T1 and L3-S1. Reflex examination was normal. His coordination was normal. Upright standing radiographs demonstrated a right thoracic curve of 20° (Figure 1A).

**Figure 3:**
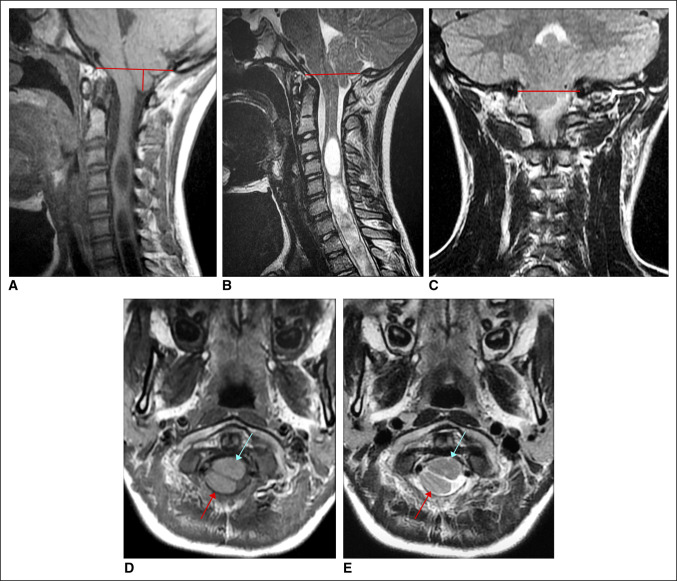
**A,** Sagittal T1 weighted MRI shows globular, mass-like hypertrophy of the right tonsil extending 11 mm below the foramen magnum (McCrae Line). Note the expansile, metameric cervical cord syrinx. **B,** Sagittal T2 weighted MRI shows globular, mass-like hypertrophy of the right tonsil extending 11 mm below the foramen magnum (McCrae Line). Note the expansile, metameric cervical cord syrinx. **C,** Coronal T2 weighted MRI shows globular, mass-like hypertrophy of the right tonsil extending 11 mm below the foramen magnum. **D,** Axial T1 weighted MRI shows inferiorly displaced right cerebellar tonsil (red arrow) posterior to the upper cervical spinal cord at the C1 level with compression along the right dorsal cord (blue arrow). **E,** Axial T2 weighted MRI shows inferiorly displaced right cerebellar tonsil (red arrow) posterior to the upper cervical spinal cord at the C1 level with compression along the right dorsal cord (blue arrow).

The patient was seen back 4 months later, at which point he was found to have further asymmetry, but no change in neurologic examination. Radiographs at that time demonstrated progression to a 26° curve (Figure 1B). Whole spine MRI was performed and showed new right-sided CM-1 with globular, mass-like configuration of the right tonsil extending 11 mm below the foramen magnum (Figure 3, A–E). The tonsil otherwise demonstrated normal signal intensity without diffusion restriction or pathologic enhancement. In addition, associated cervicomedullary junction compression (eccentric to the right Figure 3, D and E) and metameric spinal cord syrinx with cord expansion from C3 to T7 were observed (Figure 3, A and B).

**Figure 4:**
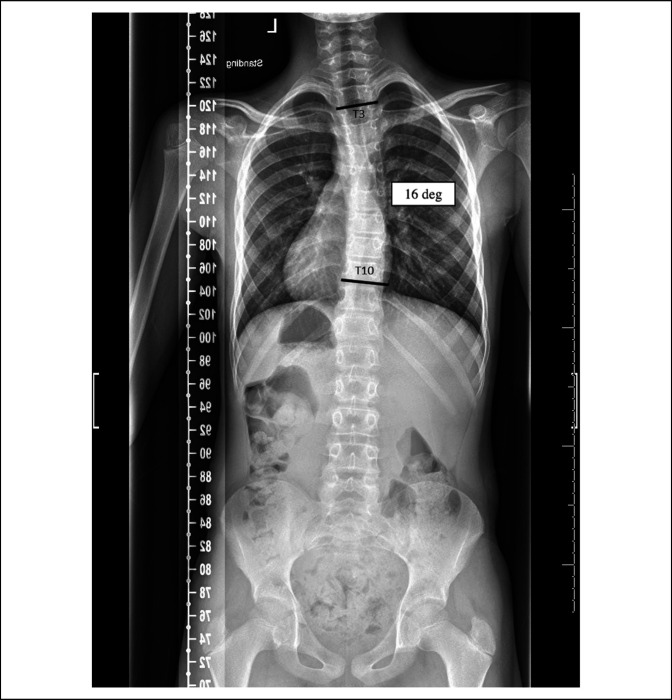
Upright standing radiograph from scoliosis series 3 months after decompressive occipital craniectomy and C1 laminectomy shows reduction of scoliosis curve with a Cobb Angle of 16°.

**Figure 5:**
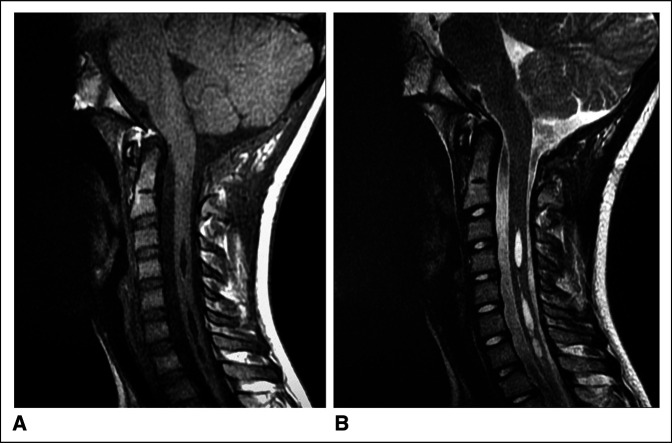
**A,** Sagittal T1 weighted MRI after occipital craniectomy and C1 laminectomy shows excellent decompression at the craniocervical junction and significant regression of the cervical cord syrinx. **B,** Sagittal T2 weighted MRI after occipital craniectomy and C1 laminectomy shows excellent decompression at the craniocervical junction and significant regression of the cervical cord syrinx.

The patient was referred to neurosurgery and underwent decompression via suboccipital craniectomy and C1 laminectomy with duraplasty. Three months postoperatively, he underwent repeat imaging with MRI of the cervical and thoracic spine and standing scoliosis radiographs (Figure 4), which showed a notable decrease in the size of the metameric syrinx (Figure 5, A and B) and reduction of the scoliosis curve to 16 ° (Figure 4). MRI demonstrated excellent decompression of the cervicomedullary junction (Figure 5, A and B).

## Discussion

CM-1 has associated syringomyelia in 30 to 70% of cases.^2^ Scoliosis is reported in over two-thirds of patients with CM-1 associated with syringomyelia.^9^ Although CM-1 was initially assumed to be predominantly congenital in etiology, the downward herniation of the cerebellar tonsils has been seen as an evolving and developing condition. In cases such as ours, patients are noted to have a normal radiological examination at birth, but tonsillar descent is observed on subsequent examinations. Studied predisposing factors relate to disproportionate posterior fossa size, mass effect from an intracranial lesion or CSF hypovolemia from chronic shunting, spontaneous CSF leak, or repeated lumbar punctures.^1,5,6^ Sometimes cerebellar tonsil enlargement is seen with reported etiologies, including neoplasm, infection, and inflammation.^7,8^

An analysis of acquired CM-1 has primarily focused on possible causative etiologies, but investigation into description of the degree, symmetry, and laterality of herniation is limited. Deng and colleagues^10^ reviewed 104 adult patients with CM-1 and syringomyelia and found that the tonsillar ectopia was unilaterally dominant in 92% and central in 8%. The side of dominance had a strong association with the side of the syrinx and the direction of curvature in those with scoliosis. Zhu et al^11^ reported on 39 patients with scoliosis and CM-1, 88% of whom had asymmetric tonsillar ectopia, 86% with same side eccentric syringomyelia, and 82% had scoliotic curves with their convex side on the same side as the tonsillar asymmetry. One study detailed the presence of dynamic cerebellar tonsils seen with asymptomatic CM-1.^12^ A retrospective study by Tubbs et al^13^ focused on the correlation between asymmetric tonsillar ectopia with unilateral symptoms and suggested that laterality is dependent on subtle differences in posterior cranial fossa morphology.

Without a definable causative factor, the underlying pathophysiology behind acquired CM-1 is poorly understood. Current hypotheses relate to changes in the absorptive pathway of CSF leading to a craniospinal pressure gradient.^14^ More research is warranted to further understand possible etiologies of acquired CM-1 in the absence of an attributable cause.

CM-1 with associated syringomyelia is the most common neurological abnormality when one is found in patients with idiopathic scoliosis despite a normal history and physical examination.^9,15^ The most common approach to management of scoliosis with CM-1 and associated syringomyelia is to address the CM-1 and syringomyelia.^4,16,17^ Decompression with the resolution of syringomyelia before correction of scoliosis is the current approach to decrease the risk of neurologic damage, but some suggest that a one-step correction of scoliosis deformity is safe in patients without preoperative neurological symptoms.^18^ As demonstrated in our case, decompression of CM-1 may also lead to the regression of a scoliosis curve. One study even showed the regression of a scoliosis curve from 54° to 4°, without separate deformity correction, once CM-1 with syringomyelia had been managed with craniocervical decompression.^15^

The rarity of our case is spontaneous CM-1 with acquired tonsillar hypertrophy of unknown etiology in a patient with a documented morphologically normal neonatal MRI Brain. The current literature does not detail acquired, spontaneous tonsillar hypertrophy and dysmorphia in CM-1. To the best of our knowledge, this is the first report of such a case and emphasizes the importance of routine MRI spine early in select patients with idiopathic scoliosis.^3,19^

## Conclusion

Spontaneous acquired type I Chiari malformation secondary to unilateral idiopathic hypertrophy of cerebellar tonsils in the absence of any known causative factor is a rare entity. The paucity of literature surrounding this case pathology indicates the need for further research into causes, management, and outcomes of spontaneous type I Chiari malformation in the absence of known etiologies. Although little is known of the exact etiology, favorable outcomes are noted after the decompressive craniectomy. As seen in this case, decompression alone may also lead to a decrease in severity of the scoliotic curve.

## Summary

Spontaneous type I Chiari malformation with acquired tonsillar hypertrophy of unknown etiology and syringomyelia supports early MRI in select patients with idiopathic scoliosis.
